# Molecular adaptations and engineering of extremophiles for synthetic biology and biotechnological applications

**DOI:** 10.3389/fmicb.2026.1754802

**Published:** 2026-02-27

**Authors:** Yusra Abdul Rehman, Amna Fayyaz, Amal Saeed Alblooshi, Khalid Muhammad, Sunil Mundra, Mohammad Tauqeer Alam

**Affiliations:** 1 Department of Biology, College of Sciences, United Arab Emirates University, Al Ain, United Arab Emirates; 2 Zayed Center for Health Sciences, United Arab Emirates University, Al Ain, United Arab Emirates; 3 Khalifa Center for Genetic Engineering and Biotechnology, United Arab Emirates University, Al Ain, United Arab Emirates

**Keywords:** biomanufacturing, climate change, extremophiles, extremozymes, genome-scale metabolic modeling, metabolic engineering, synthetic biology

## Abstract

Extremophiles are microorganisms that thrive in environments previously thought to be uninhabitable, including extreme temperature, salinity, pH, pressure, and radiation. These organisms, found in Archaea, Bacteria, and Eukarya, exhibit distinct structural, metabolic, and genetic adaptations, such as enhanced enzyme stability, efficient DNA repair mechanisms, and robust stress-response systems that enable survival under extreme conditions. Understanding these adaptation mechanisms is key to engineering similar traits in mesophilic organisms. This review discusses the diversity of extremophiles and presents phylogenetic and comparative genomic insights which may provide insights into the origins and evolution of early life on Earth We highlight recent advances in CRISPR/Cas-based genome editing, genome-scale metabolic modeling (GEM), and synthetic biology that have expanded the use of extremophiles in sustainable industrial biotechnology. The exceptional stability and catalytic efficiency of extremozymes under harsh conditions underscore their potential in various biotechnological applications. Finally, we discuss the ecological significance of extremophiles in climate change mitigation and outline current challenges and future directions in extremophile research.

## Introduction

Microorganisms inhabit nearly every environment on Earth (Thompson et al., 2017). Those capable of surviving under extreme conditions such as high or low temperatures, extreme pH, high salinity, or intense radiation are known as extremophiles (Zhao et al., 2010). These organisms are primarily found within the domains *Archaea* and *Bacteria*, although certain eukaryotic species have also been identified as extremophiles. Extremophiles exhibit remarkable adaptability in environments once considered uninhabitable, ranging from the scorching heat of hydrothermal vents to the acidic conditions of volcanic springs (Das and Dash, 2018). They possess unique physiological and biochemical adaptations that enable them to survive and even thrive under such hostile conditions. These adaptations include robust DNA repair systems that counteract radiation damage, stress-resistant membrane structures and lipid compositions, accumulation of organic osmolytes, production of specialized enzymes, and protein-level modifications that maintain intracellular homeostasis (de Lours Moreno et al., 2013).

Extremophiles represent a unique group of organisms with broad relevance to biotechnology. In the field of biotechnology, these organisms are renowned for their robust enzymes, thereby serving as invaluable resources for a multitude of industrial processes. Furthermore, these extremophilic enzymes are used across a wide range of industries, including biofuels, pharmaceuticals, and environmental remediation (Abe and Horikoshi, 2001). Moreover, using computational and synthetic biology approaches it is possible to understand the mechanism of adaptation, as well as the production of important compounds. Additionally, the discovery of extremophiles has ramifications for the field of astrobiology, suggesting that life could survive in extreme extraterrestrial environments and will help in understanding the limits of life and life on other planets (Rampelotto, 2010).

This review provides an overview of the diversity and evolutionary relationships of extremophiles, followed by a discussion of the structural and functional adaptations of extremozymes and their industrial and biotechnological applications. Furthermore, the review explores the role of extremophiles in climate change mitigation and adaptation, and concludes by outlining current challenges and future directions in extremophile research.

## Diversity of extremophiles

**Figure 1:**
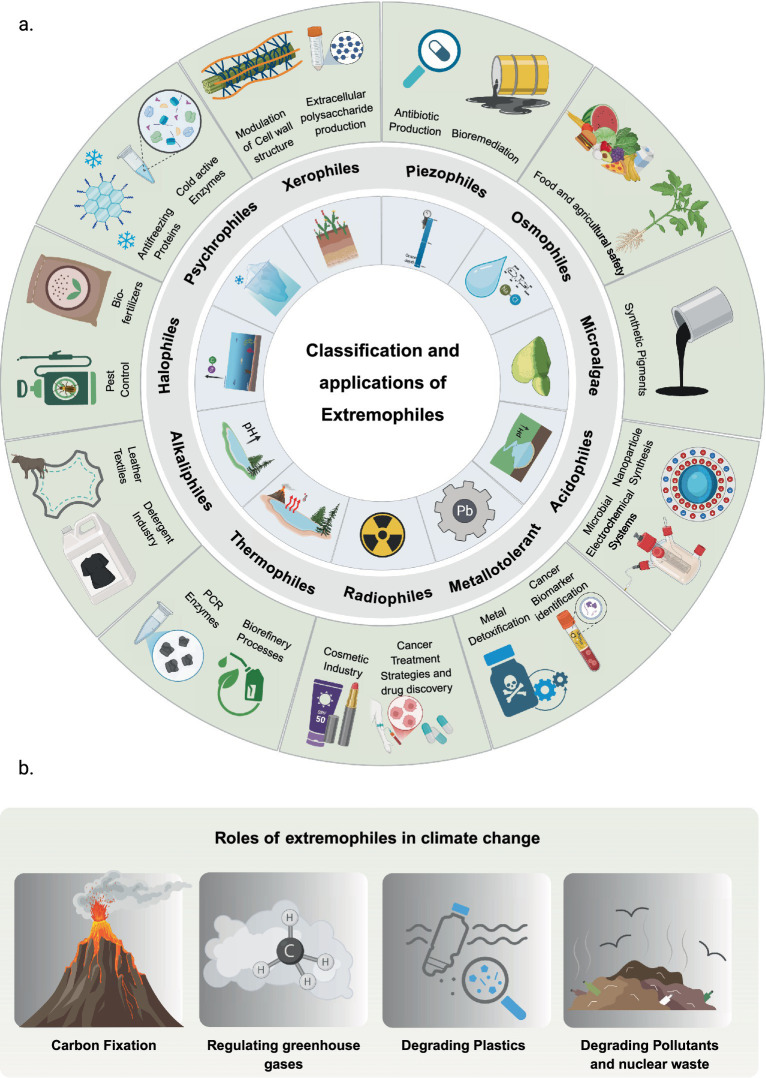
Industrial applications and role in the climate change of different classes of extremophiles. **(a)** Illustrations in the inner circle are representing the major classification of extremophiles, while the outer circle depicts their associated industrial and biotechnological applications. **(b)** Representation of key mechanisms by which extremophiles contribute to climate change mitigation and environmental sustainability, highlighting processes that can be harnessed through synthetic biology and metabolic engineering approaches.

Extremophiles are diverse microorganisms that thrive in extreme environments, from hydrothermal vents and polar ice caps to acidic pools, alkaline lakes, hypersaline waters, ocean depths, and radiation-exposed regions like Death Valley, California (Rothschild and Mancinelli, 2001). Their remarkable diversity is reflected in the specialized metabolic pathways (Alblooshi et al., 2025), cellular structures, and genetic adaptations that allow these organisms to harness energy, sustain metabolic processes, and preserve genomic integrity in environments that would be lethal to nearly all other known forms of life (Tse and Ma, 2016; Alblooshi et al., 2025). Based on their habitats, these species are categorized into diverse groups, including Thermophiles, Psychrophiles, Halophiles, Piezophiles, Acidophiles, and Radiophiles (Figure 1a).

Extremophiles adapted to extreme temperature are broadly categorized into two major groups: thermophiles [above 50 °C], usually located in hot springs and hydrothermal vents (Lundgren and Bernander, 2005), and psychrophiles [below 15 °C], more abundant in marine habitat (Deming, 2002; De Maayer et al., 2014; Urbanek et al., 2018). Thermophiles contain stable enzymes that resist heat and special features like temperature-responsive membrane lipids, durable cell membranes, and elevated GC levels in rRNA and tRNA to improve molecular stability (Counts et al., 2017; Alblooshi et al., 2025). Likewise, Psychrophiles exhibit distinct molecular and genomic features including flexibility in their enzymatic activity, a higher number of antifreeze and cold-shock proteins, membrane fluidity, decreased hydrogen bonding and greater hydrophobicity on their surface which help them to survive in the cold environments (Guan et al., 2013; Alblooshi et al., 2025). The Antarctic ciliate *Euplotes focardii* and its associated bacterial consortium represent a well-studied psychrophilic system, showing pronounced molecular adaptation for cold, which identified cold-active lipases with high efficiency at low temperature. In addition, metagenomic studies also identified the antifreezing protein (EfcIBP), which provides strong cryoprotection, underscoring the cooperative survival strategy of the consortium in Antarctic environments (Pucciarelli et al., 2015; Mangiagalli et al., 2017).

Another major group of extremophiles is the halophiles, which thrive in hypersaline environments through specialized adaptations such as salt-stable proteins and enzymes (Lanyi, 1974), accumulation of compatible solutes like potassium ions and glycine betaine (Roberts, 2005), and modified cell membranes coupled with efficient DNA repair mechanisms (Jones and Baxter, 2017). Other extremophile groups include acidophiles, alkaliphiles, piezophiles, and radiophiles each adapted to survive in unique extreme environments. Acidophiles inhabit highly acidic environments with pH levels below 3 and survive by maintaining near-neutral internal pH, producing acid stable enzymes, and strengthening their membranes (Méndez-García et al., 2015; Quehenberger et al., 2017; Aguilera and González-Toril, 2019; Ianutsevich et al., 2023). Alkaliphiles, which thrive in high pH environment (Horikoshi, 1999); Xerophiles, adapted to arid conditions; Metallotolerant, capable of detoxifying heavy metals; and Osmophiles, which maintain osmotic balance in saline or sugary habitats (Rao et al., 2022; Parades-Aguilar et al., 2024). Piezophiles thrive under immense deep-sea pressure through flexible membranes and pressure resistant enzymes that maintain cellular function (Meersman et al., 2013; Picard and Daniel, 2013; Cario et al., 2019; Tamby et al., 2023). *Radiophiles* endure extreme radiation through efficient DNA repair, antioxidant defenses, and radiation-tolerant proteins (Butterworth et al., 2023; Khan et al., 2024).

## Evolutionary relationship of extremophiles

The Last Universal Common Ancestor (LUCA) refers to an ancestral organism from which all known modern organisms have evolved. It likely existed billions of years ago, during a period when the Earth itself was characterized by extreme and life-limiting environmental conditions, therefore LUCA was an extremophile (Ali et al., 2023). The LUCA likely endured intense heat and anaerobic conditions similar to those of modern extremophiles, supporting theories that early life was thermophilic or hyperthermophilic (Coker, 2023). Moreover, studies support the idea that the LUCA shared characteristics with modern thermophiles and hyperthermophiles and that many of the adaptations to extreme conditions arose early in life’s evolutionary history (Bhattacharya et al., 1999). Furthermore, comparisons of ribosomal RNA (rRNA) sequences, heat-shock proteins, and membrane lipid compositions provide insights into the evolutionary divergence of extremophiles from their common ancestors (de la Haba et al., 2022).

However, it is important to note that extremophiles are found across all three domains of life including Bacteria, Archaea, and Eukarya, sharing similar mechanisms that enable survival in extreme environments. This broad distribution indicates that extremophilic features cannot be attributed solely to direct inheritance from the last universal common ancestor (LUCA). Instead, these shared traits likely arose through convergent evolution and/or the horizontal acquisition of stress-resistance genes from a common environmental gene pool via horizontal gene transfer (HGT) (Gallo and Aulitto, 2024). The acquisition of such crucial genes is postulated to have been the principle key for the evolutionary success of extremophiles providing them with survival attributes under extreme environmental conditions. For example, genes that provide for resistance to high temperature and radiation may have been transferred between early archaea and bacteria (Rampelotto, 2024). The biochemical activity of these proteins likely enhanced genetic exchange and thus the existence of multiple extremophilic lineages throughout a variety of environmental niches (Cox and Battista, 2005). As a result, the evolutionary history of extremophiles is less linear (not solely vertical inheritance) and more quilted. Subsequent horizontal gene transfer events such as these have undoubtedly aided to this remarkable degree of adaptability among extremophiles, complexifying the perception of evolutionary ties between them and emphasizing seminal function HGT played in their harsh-environment prosperance (Rampelotto, 2024).

Furthermore, regarding the evolutionary relationships among groups of extremophilic organisms, extremophilic bacteria exhibit significant environmental adaptations shaped by evolution. Notable examples include the thermophilic bacterium *Thermus aquaticus*, instrumental in the development of PCR technology, and *Deinococcus radiodurans*, renowned for its exceptional resistance to radiation and desiccation (Cox and Battista, 2005). The Last Universal Common Ancestor (LUCA) of most extremophilic bacteria likely inhabited similar extreme environments, suggesting that early life may have evolved under conditions resembling those in which LUCA and its descendants survived (Moody et al., 2024). The genus *Thermus* represents an ancient bacterial lineage that diverged early and retained its capacity to thrive at high temperatures. This heat tolerance likely reflects a long evolutionary history tracing back to the common ancestor of extremophilic bacteria, contributing to *Thermus* species’ ability to colonize hot geothermal springs (Wang et al., 2021). Similarly, the remarkable radiation resistance of *Deinococcus* is thought to have arisen as an adaptation to the harsh conditions of early Earth, characterized by intense radiation and frequent environmental stresses. The combined resistance of *Deinococcus* to radiation, desiccation, and oxidative damage underscores its evolutionary adaptation to such extreme environments. Collectively, these traits illustrate how environmental selective pressures have driven the evolution of resilience in extremophilic species (Toueille and Sommer, 2011).

Lastly, the enzymes of extremophilic species, known as extremozymes, are specifically adapted to function under extreme environmental conditions through unique structural and functional modifications that enable them to maintain activity in such habitats (D’Amico et al., 2003). Consequently, the ability of extremophiles to survive in extreme environments is largely attributed to the evolutionary development of these extremozymes, driven by natural selection, gene duplication, and horizontal gene transfer (Gallo and Aulitto, 2024).

## Structural and functional adaptations of extremozymes

Extremophile-produced enzymes, known as ‘extremozymes,’ can withstand extreme conditions such as high temperature, high salt concentration, and high pressure outperforming normal enzymes that degrade in such environments (Table 1) (Hough and Danson, 1999). The protein stability in extremozymes is due to having a larger number of ionic bonds, hydrophobic interactions, and hydrogen bonds which contribute in powering the enzymes overall structure (Jaenicke, 1991). This strengthening prevents enzymes from denaturing or losing its functional shape under stressful conditions (Scandurra et al., 1998). Moreover, extremozymes have specific amino acids that increase their stability in extreme conditions (Alblooshi et al., 2025).

**Table 1:** Extremophile diversity and their associated enzymes.

Extremophiles	Optimal conditions	Examples	Extremozymes	References
Thermophiles	High temperature (above 50 °C)	*Thermus aquaticus*, *Pyrococcus furiosus*	Lipases, laccases, xylanase, polymerase	Jin et al. (2019), Sysoev et al. (2021)
Psychrophiles	Low temperatures (−20 to 20 °C)	*Pandalus borealis*, *Euphausia superba*, *Moraxella species*, *Flavobacterium species*, *Euplotes focardii*	Xylanase, protease, esterase, b-glycosidase, lipases, alpha amylase	Yang et al. (2017, 2021), Jin et al. (2019), Sysoev et al. (2021), Liu et al. (2022)
Halophiles	High salt (2 to 6 M NaCl)	*Naloterringena hispanica*, *Natronococcus occultus*, *Halobacterium*, *Haloferax*	Amylase, protease, xylanase, esterase, nucleases, cellulases, chitinases, alcohol dehydrogenases, lipases	Jin et al. (2019), Sysoev et al. (2021)
Acidophiles	Low PH (<3)	*Penicillium* spp., *Sulfolobus solfactaricus*	Matrix metallopeptidase inhibitor, trehalase, proteolytic enzymes	Jin et al. (2019), Sysoev et al. (2021)
Alkaliphiles	High PH (>9)	*Alkalibacillus* sp.	Cellulase, esterase, serine protease	Jin et al. (2019), Sysoev et al. (2021)
Radiophiles	High radiation (gamma/UV/X rays)	*Porphyra rosengurttii*, *Deinococcus radiodurans*	Deinoxanthin, bacteriorubein, mycosporin-like amino acids	Sysoev et al. (2021), Mesbah (2022)
Polyextremophiles	Different extreme condition (like, temperature and salt)	*Halothermophiles, Halopsychrophiles*, *Alkalibacillus* sp. *NM-Da2*	Alkalithermophilic serine proteases, alkalipsychrophilic esterase	Jin et al. (2019)

Mesophilic enzymes are optimized for moderate environmental conditions. Within a narrow range of temperatures, neutral pH, and normal atmospheric pressure. They lack stabilizing interactions, making them more prone to denaturation, which makes them more susceptible to denaturation when exposed to extreme environments. Thermophilic species exhibited a marked enrichment of amino acids such as tyrosine, glutamate, and leucine, while showing significantly lower levels of cysteine, alanine, arginine, glutamine, and asparagine. In contrast, psychrophilic species displayed elevated concentrations of threonine, methionine, phenylalanine, serine, and tyrosine but reduced levels of asparagine, arginine, alanine, cysteine, and proline (Alblooshi et al., 2025). Thermostable DNA polymerases represent a classic and transformative example of extremozymes, such as Taq polymerase from *Thermus aquaticus*, Pfu polymerase from *Pyrococcus furiosus*, and KOD polymerase from *Thermococcus kodakarensis*, retains high catalytic activity at elevated temperatures and enables efficient PCR amplification, outperforming mesophilic polymerases that denature during thermal cycling (Cline et al., 1996; Yamashita et al., 2017; Turvey et al., 2022). Similarly, Halophilic enzymes have an abundance of acidic amino acids which contribute in enhancing the solubility and preventing the aggregation in saline environments (Pan et al., 2020).

In terms of the surface charge and hydrophobicity, depending on the environmental stress, extremozymes may show altered surface charge distributions or increased hydrophobicity (Salas-Bruggink et al., 2024). For instance, piezophilic enzymes usually have more charged amino acids on their surfaces to counteract the effects of high pressure, which can compress proteins and disrupt their function (Nath and Subbiah, 2016). On the other hand, mesophilic enzymes do not require any specialized surface properties, as they live in environments where pressure, salinity, and pH are stable and moderate (Rabbani et al., 2023).

The gene sequences of extremozymes are generally found to have some mutations that can be seen as specific changes in their code; these modifications improve enzyme structure and function under extreme conditions. These mutations can be due to alterations in the primary amino acid sequence that impact enzyme folding and stability (Jin et al., 2019). In these cases, the extremozymes are often paired with regulatory elements to prevent wasteful expression of such enzymes under standard environmental conditions (Grünberger et al., 2023).

Enzyme expression is regulated by environmental factors such as temperature and pH, and other moderate environmental conditions. Extensive efforts have been made to isolate and characterize the halophilic enzymes from salt brine, marine environments, the Dead Sea, and hypersaline soda lakes (Karan et al., 2012b; Ventosa et al., 2015). A recent report shows isolated strains of bacteria, archaea, and fungi by culture-based approaches were found to secrete hydrolases (protease, lipase, amylase, cellulase, xylanase, and pectinase) using agar plate-based assays (Ruginescu et al., 2020). Applications include enzymes, compatible solutes, biopolymers, and more (Moreno et al., 2020).

## Industrial and biotechnological application of extremophiles

Extremophiles possess unique biochemical traits that have advanced multiple industrial fields, including agriculture, textile and leather industries, detergents, biofuel, drugs and cosmetology, biorefinery, material science, basic science research, and food and beverages (Figure 1a) (Cabrera and Blamey, 2018). In the field of agriculture, extremophiles are associated with their use as biofertilizers, improving soil fertility and plant growth even under harsh conditions like saline or acidic soils devoid from nutrients (Nweze et al., 2022). Extremophilic bacteria that can fix nitrogen convert atmospheric nitrogen into available forms for plants and by association enhance productivity in previously unproductive zones such as rice or legumes (Mahmud et al., 2020). Furthermore, acidophilic and halophilic microorganisms are good at solubilizing insoluble phosphate compounds leading to an enhancement in the available phosphorus pools of wheat-barley plants (Timofeeva et al., 2022). These extremophiles function together to aid in better plant growth and agriculture productivity on challenging soil conditions (Zenteno-Alegría et al., 2024). Moreover, the growing incorporation of extremophiles as biocontrol agents in agriculture for disease and pest management offers a sustainable alternative to chemical pesticide use (Mattedi et al., 2023). Assortments of antifungal molecules which rule out harmful pathogens are bioavailable compounds secreted by various groups of microorganisms (Brauer et al., 2019).

Regarding textile and leather industries, extremophiles are used for various processes including desizing, scouring and bleaching of fabrics. Very often these steps involve high temperatures and harsh chemicals to strip out all the impurities which create a great medium for dyeing (Panda et al., 2024). Thermophiles and alkaliphiles show improved activity for these kinds of modifications without a concomitant increase on water and energy costs associated to the reaction conditions nor with the amount of chemicals that are necessary in this process (Gallo and Aulitto, 2024). Extremozymes are utilized in the leather industry to improve the tanning process. Extremophilic proteases are applied to dehairing and bating steps of leather processing; a method which uses enzymes degradation (Dayanandan et al., 2003).

Extremophilic species have made a revolutionary change in the detergent industry (Arora et al., 2022). For example, a significant type of extremozymes found in commercial detergents consist of those obtained from alkaliphilic microorganisms (Ito et al., 1998). The alkaliphilic serine proteases belong to a group of enzymes of this kind that are widely used, as they break down protein-based stains such as blood, sweat and various food residues at high pH values (Yang et al., 2024). Additionally, due to the predominantly alkaline conditions for laundry detergents, these enzymes contribute significantly to stain removal in cold water washes. This helps reduce energy use in washing processes if consumers are able to wash their clothes by using cold or warm water instead of hot water (Qin et al., 2014).

Further, in the global effort to develop sustainable biofuel energy, extremophiles have been increasingly identified by biofuel entrepreneurs as key players (Gallo and Aulitto, 2024). Extremophiles produce enzymes and metabolic pathways for the decomposition of biomass and biofuel production under typical processes which would generally denature conventional proteins. Such an exclusive property makes them essential for biohydrogen, biogas, and bioethanol, which are eco-friendly substitutes for conventional fossil fuels (Datta et al., 2019). One of the most interesting uses of extremophiles for biofuel production is in producing biogas by anaerobic digestion (Archana et al., 2024). In this process extremophile organisms like thermophiles, operate at high temperatures, enabling faster degradation of organic waste including agricultural wastes, animal manure, municipal solid waste (Neri et al., 2023). Achieving thermophilic conditions (at typically 50–70 °C) will lead to the high efficiency of anaerobic digestion that gives higher methane yields in biogas production (David et al., 2018).

Toward the development of various drugs by producing unique bioactive compounds and enzymes, extremophiles have been very useful. These organisms represent a potential source of novel antibiotics, antiviral drugs and anticancer agents. Whereas these compounds show significant activity against the drug-resistant pathogens (Baranova et al., 2020). For example, extremozymes are employed in the synthesis of drugs for this is a result of their stability and efficiency under harsh conditions which leads to an improvement on production level (Niehaus et al., 1999). Moreover, extremophilic proteins and lipids are also investigated for drug stability and delivery, which signifies their prospective roles as strategic moieties of biopharmaceuticals (Kumar et al., 2018). These species are increasingly being utilized in the cosmetics industry for their unique biochemical properties. They are valuable in skincare and personal care products. These organisms produce compounds that offer exceptional stability and efficacy under harsh conditions, which makes them ideal for UV protection, anti-aging, moisturization, and skin barrier protection (Sepe et al., 2025). For Example, extremophiles from high-radiation environments produce mycosporine-like amino acids (MAAs). They are effective UV-absorbing compounds and are used in sunscreens and anti-aging creams (Sinha and Häder, 2008). Additionally, extremophiles that thrive in high salinity produce molecules like ectoine and exopolysaccharides, which help retain moisture, strengthen the skin barrier, and soothe the irritated skin (Ma Z. et al., 2022).

Furthermore, extremophiles are important sources in integrated biorefinery systems, which are conceptualized as facilities to convert biomass into products such as biofuels, biochemicals, and biomaterials (Gallo and Aulitto, 2024). Recently, extremophiles gained much attention in the field of material science because they produce novel biomolecules, such as proteins, polysaccharides and lipids which survive at high temperatures or pH values (Gallo and Aulitto, 2024). These species are important in the development of nanomaterials (Chauhan et al., 2023). They provide a new source for the synthesis of nanoparticles and nanostructures, which can help replace traditional and toxic methods of nanoparticle synthesis with sustainable ones (Said et al., 2023). Some thermophilic and halophilic microorganisms can synthesize metallic nanoparticles such as gold, silver, and titanium oxide from metal ions through biological processes that are both eco-friendly and energy-efficient (Said et al., 2023). Nanoparticles derived from extremophiles have a range of technological applications such as antimicrobial coatings, and catalysts for chemical reactions and optical devices (Erkoc and Ulucan-Karnak, 2021).

## Mathematical models of extremophiles

**Figure 2:**
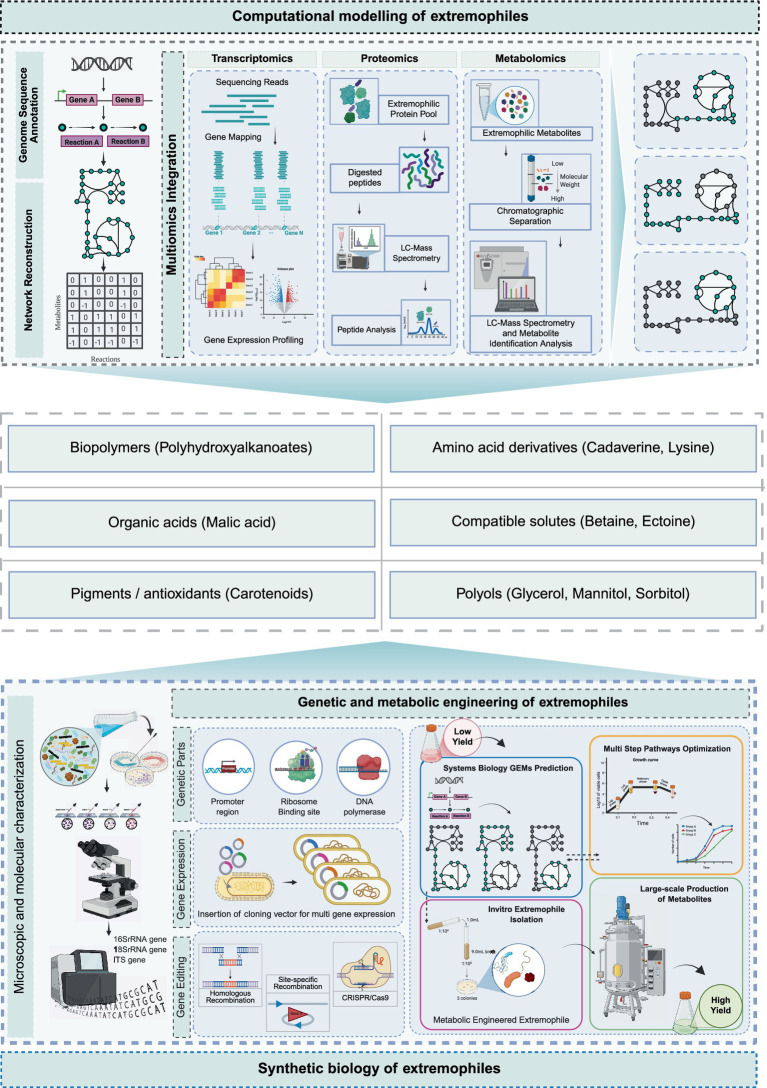
Integration of computational modeling and genetic and metabolic engineering of extremophiles to generate synthetic biomolecules. The top panel is representing the combination of multi-omics data to reconstruct genome-scale metabolic models of extremophiles at a systems-level understanding. The bottom panel illustrates how insights from these models inform synthetic biology and metabolic engineering strategies for the efficient production of industrially relevant biomolecules.

One of the major challenges in laboratory studies of extremophiles is replicating their natural extreme environments to enable successful cultivation. This limitation has contributed to the relatively limited research and understanding of these highly intriguing organisms. However, genome-scale metabolic models (GEMs) have been widely applied to investigate mechanisms of microbial adaptation under various environmental conditions (Carter et al., 2024b). The integration of multi-omics datasets into these computational frameworks has further enhanced our understanding of microbial diversity (Carter et al., 2024a), facilitating advances in metabolic engineering (Figure 2) (Kim et al., 2025), drug target discovery (Rienksma et al., 2014), and studies of adaptive responses to environmental change (Ates et al., 2011; Fondi et al., 2015).

GEMs of extremophiles provide valuable insights into the diverse strategies these organisms employ to survive in harsh environments (Carter et al., 2024a; Wutkowska et al., 2024). This approach complements genomic and physicochemical analyses and is essential for interpreting specialized proteins, such as heat-shock and ice-binding proteins (McCallum et al., 1986; Rappaport and Oliverio, 2024). For example, mesophilic organisms can adapt to nonoptimal growth temperatures by expressing cold- or heat-shock proteins (Zhou et al., 2021) and by modifying protein synthesis and its disruption (Dufault-Thompson et al., 2022). In contrast, psychrophiles constitutively produce proteins typically associated with cold-shock responses (Zeng et al., 2016). Comparative GEM analyses among thermophiles, mesophiles, and psychrophiles have revealed that temperature adaptation is reflected in genome size, GC content, and metabolic network architecture (Hu et al., 2022; Steindorff et al., 2024; Alblooshi et al., 2025). Psychrophiles generally possess larger genomes with more coding genes, whereas thermophiles exhibit smaller genomes but denser metabolic networks (Wang et al., 2015; Alblooshi et al., 2025). GEMs have also uncovered specialized pathways and coenzyme affinities that enhance energy production under extreme conditions (Karan et al., 2012a). Furthermore, these models have been instrumental in examining horizontal gene transfer, DNA repair, and regulatory mechanisms that enable extremophiles to maintain homeostasis in their habitats (Dufault-Thompson et al., 2017).

Mathematical model–based cross-environmental comparisons further allow uniform evaluation of metabolic networks, facilitating the identification of conserved adaptive strategies among species and environments (Bräsen et al., 2014; Alblooshi et al., 2025). Recent advances in systems biology have enabled the reconstruction of comprehensive metabolic networks, revealing extensive regulatory crosstalk between pathways and highlighting the importance of enzyme–metabolite interactions in metabolic control. For example, integrating genome-scale metabolic models with cross-species enzyme kinetic data has uncovered widespread regulatory interactions that influence network behavior and metabolic adaptability in *S. cerevisiae* (Al Zubaidi et al., 2025). In applied contexts, such models can define measurable intervention strategies aimed at optimizing ATP production or enhancing the biosynthesis of thermostable enzymes (extremozymes). For example, growth-coupled overproduction has been computationally demonstrated in various organisms, where restricting metabolites such as enzyme precursors or ATP drives targeted overproduction through specific gene knockouts (von Kamp and Klamt, 2017). Thermophiles have shown the ability to overexpress and produce stable enzymes (e.g., Mn-dependent catalases from *T. thermophilus*), while designed *P. furiosus* models facilitate flux rerouting to enhance reductant and ATP supply for specific pathways under high-temperature conditions (Hidalgo et al., 2004). In context-specific GEMs, we typically identify the optimal combination of reaction fluxes representing the most efficient steady state of a cell—usually one that maximizes or minimizes a defined objective function such as growth rate (Figure 2) (Moyer et al., 2025).

In natural environments, extremophilic microorganisms often exist within complex microbial consortia, where metabolite exchange and cooperative interactions are essential for survival (Sabih Ur Rehman et al., 2025). Modeling these communities enables examination of both interspecies interactions and environmental responses. A recent study demonstrated that extremophilic species generally exhibit fewer interactions with their environment compared to mesophiles, with psychrophiles importing fewer nutrients and thermophiles exporting fewer metabolites (Alblooshi et al., 2025). Community-scale GEMs and syntrophic flux modeling allow the simulation of finding the way species exchange hydrogen, formate, acetate, or other intermediates, and partition metabolic tasks for sustaining growth, which is infeasible in an isolated environment (Nagarajan et al., 2013; Diener and Gibbons, 2023; Tanniche and Behkam, 2024). These models have been successfully applied to dissect metabolic exchanges and division of labor in extreme ecosystems. For instance, in a hot-spring temperature gradient, a study has reconstructed GSMMs for every metagenome-assembled genome (MAG) and computed a metabolic complementarity index to map cross-feeding and pinpoint archaeal hubs (i.e., *Thermoproteota* and *Methanobacteriota*) that organize thermophilic networks (Peng et al., 2024). Similarly, modeling of a hypersaline archaeon–bacterium consortium (*Halorubrum* sp. and *Marinococcus luteus*, ~25%) revealed minimal media requirements, essential nutrients, and bidirectional metabolite exchange underlying mutual dependence (Medina-Chávez et al., 2023). Overall, predictions derived from such genome-scale and community-level models are invaluable for advancing our understanding of microbial behavior, adaptation, and ecosystem-level interactions among extremophilic species in their natural habitats.

## Genetic and metabolic engineering of extremophiles using synthetic biology approaches

Conventional chemical engineering processes used in industrial production have been linked to various environmental disadvantages, such as the emission of carbon dioxide, diminished sustainability, dependence on non-renewable resources, and heightened pollution from particulate and chemical contaminants (Clomburg et al., 2017). To address these issues, microorganisms are increasingly being utilized as biomanufacturing systems for the safe and sustainable production of various chemicals without harmful environmental effects (Philp et al., 2013). In this context, synthetic biology has emerged as a key driving force in biomanufacturing, enhancing the biosynthesis of valuable products such as bioplastics, food additives, biofuels, and other industrially relevant chemicals (Steen et al., 2008; Chen et al., 2015; Fang et al., 2018).

Current industrial biotechnology primarily relies on traditional microbial hosts such as *Saccharomyces cerevisiae* (yeast), *Escherichia coli*, *Pseudomonas* spp., *Ralstonia eutropha*, and *Bacillus* spp. However, these systems are often limited by factors such as long cultivation times, high production costs, microbial contamination, intensive water and energy demands, difficulties in large-scale cultivation, and complex downstream processing workflows (Chen et al., 2015). To overcome these challenges, next-generation industrial biotechnology has increasingly turned to extremophiles as alternative microbial platforms. Through advanced molecular engineering approaches and the use of specialized genetic tools, extremophilic microorganisms can be optimized for efficient bioproduction, reducing contamination risks, energy consumption, process complexity, and carbon emissions. Among these, *Halomonas* spp. have emerged as promising candidates due to their ability to grow at high pH (8–10) and elevated salinity (30–80 g/L NaCl), enabling cost-effective, open, and continuous fermentation processes (Chen and Jiang, 2018; Bonnaud et al., 2024). Similarly, *Haloferax bluephagenesis* TD01, which grows under high salinity and alkaline conditions, is a well-established archaeal production platform, particularly for the biosynthesis of polyhydroxyalkanoates (PHAs/PHB) and other bioproducts (Xu et al., 2022). In addition, alkaliphilic microorganisms have been explored for biofuel and chemical production; *Clostridium alkalicellulosi* has been studied for the production of hydrogen, acetate, lactate, and ethanol, while alkaliphilic methanogenic archaea such as *Methanosaeta* and *Methanocalculus* species have been applied in methane-producing bioprocesses, primarily through process optimization rather than molecular engineering (Sousa et al., 2015).

Metabolic engineering of extremophiles remains a challenging yet promising application in industrial biotechnology. To address issues of metabolic instability and improve production efficiency, several molecular and genetic strategies have been developed (Zheng et al., 2025). These include the optimization and engineering of promoters (Li et al., 2016; Shen et al., 2018) and enzymes (Lan et al., 2016), adaptive gene regulation (Gupta et al., 2017; Ma Y. et al., 2022), optimization of ribosome binding sites (Shi et al., 2018; Stiller et al., 2018), and multiple expression of genes and their pathways (Ye et al., 2023). Multiple gene expression plasmids have been constructed, particularly for *Pseudomonas* and *Halomonas* spp., yet maintaining plasmid stability and transformation efficiency in extremophiles remains difficult due to host restriction–modification systems (Lammens et al., 2022; Ye et al., 2023). For instance, in *Clostridium thermocellum*, host restriction enzymes can degrade unmethylated DNA at GATC sites, thereby reducing transformation efficiency (Klapatch et al., 1996). Recent advances in genome-editing technologies, including CRISPR/Cas9, have expanded the genetic toolkit available for extremophiles, enabling precise site-specific mutagenesis through gene knock-in and knockout approaches in organisms such as *Kluyveromyces marxianus*, *Clostridium cellulolyticum*, and *Clostridium thermocellum* (Xu et al., 2015; Löbs et al., 2017; Walker et al., 2020). Similarly, the thermophilic anaerobe *Bacillus coagulans* has been engineered via homologous recombination to delete genes associated with competing metabolic pathways, resulting in enhanced malic acid production (Figure 2) (Sun et al., 2021). In parallel, CRISPR/Cas-based genome-editing systems have been developed for archaeal species such as *Haloferax volcanii* and *Haloferax salinarum*, enabling precise genetic manipulation and facilitating studies of stress tolerance and metabolic regulation under high-salinity conditions. Collectively, these advances support the development of extremophilic microbial platforms for industrial bioprocessing and lay the groundwork for future bioproduct synthesis (Qin et al., 2018).

Through these molecular approaches, extremophilic cells can be developed as efficient microbial factories for the synthesis of high-value bioproducts. Metabolic engineering efforts have successfully targeted the production of enzymes, polyhydroxyalkanoates (PHAs), cadaverine, polyols, amino acids, betaine, and ectoine (Figure 2) (Tan et al., 2014; Zhao et al., 2019; Ma et al., 2020; Liu et al., 2022). For example, to produce cadaverine—a key precursor for nylon—the *ldcC*-linked lysine decarboxylase gene from *E. coli* was introduced into the haloalkaliphilic bacterium *Halomonas campaniensis* LC-9, enabling *de novo* synthesis of the compound (Zhao et al., 2022). Several *Halomonas* species, including *Halomonas* sp. HAL1, *H. bluephagenesis*, *Halomonas* sp. KM-1, and *H. campaniensis* LS21, have demonstrated the ability to synthesize PHAs efficiently under alkaline and high-salt conditions. Accumulation of PHA from industrial waste and cost-effective carbon sources has been associated with halophilic archaea (Koller, 2019; Obruča et al., 2022). Likewise, the halophilic bacterium *Vibrio natriegens* has been engineered to reduce energy and freshwater consumption, facilitating the low-cost production of various metabolic targets (Ye and Chen, 2021; Meng et al., 2022). Additionally, several extremophiles, including halophiles from Letea Lake, *Thermus thermophilus*, and *Deinococcus radiodurans*, naturally produce carotenoids with potent antioxidant properties (Ram et al., 2020). Extremophiles also include radioresistant archaea that have ability to grow under gamma, X and UV radiations which is helpful for bioremediation near nuclear power plants that cannot be done by common (in use) microorganisms. During the exposure of radiations these radioresistant organsims protect their cells from reactive oxygen species (ROS) by the collection of biocompatible solutes, specific proteins and pigments inside their cells to overcome any oxidative damage (Gabani and Singh, 2013). In summary, extremophiles offer a promising foundation for next-generation industrial biotechnology, combining resilience with metabolic versatility. Advances in engineering and synthetic biology now enable their use in sustainable bioproduction, reducing environmental impact while enhancing process efficiency.

## Extremophile’s role in climate change mitigation and adaptation

Extremophiles play an important role in the context of climate change, particularly through their contribution to the global carbon cycle with increasing relevance for extremophile-based biotechnological applications (González and Terrón, 2021). Many chemolithoautotrophic organisms found in extreme environments such as hydrothermal vents and cold seeps contribute to carbon fixation by utilizing inorganic compounds like methane to convert carbon dioxide into organic matter (Dang and Chen, 2017). In doing so, these microorganisms help regulate global carbon levels, a process critical to maintaining Earth’s climate balance (Offre et al., 2013). Studying extremophiles improves our understanding of life’s adaptability under extreme stress, which is a topic of growing relevance as climate change drives environments toward higher temperatures, increased salinity, and ocean acidification. Insights into the adaptive mechanisms of extremophiles may inform broader biological strategies for resilience under changing environmental conditions (Figure 1b) (Stetter, 1999).

Moreover, extremophiles influence atmospheric greenhouse gas concentrations, thereby impacting climate regulation (González and Terrón, 2021). Methanogens, which are extremophilic archaea that produce methane from carbon dioxide and hydrogen, contribute significantly to global methane emissions, a potent greenhouse gas. Conversely, methanotrophs, which thrive under similar extreme conditions, consume methane, thereby reducing its atmospheric levels. The interplay between methane-producing and methane-consuming microbes plays a key role in maintaining Earth’s greenhouse gas equilibrium and engineering these extremophilic methane-cycling pathways offers promising opportunities for biotechnological strategies aimed at mitigating greenhouse gas emissions (Marlow et al., 2014).

Extremophiles also hold great potential for environmental remediation. Certain plastic-degrading bacteria, such as *Pseudomonas* spp. and *Ideonella sakaiensis*, can break down synthetic polymers like polyethylene terephthalate (PET) (Freund et al., 2025). *I. sakaiensis* produces the enzyme PETase, which hydrolyzes PET into its monomeric components, facilitating natural degradation and significantly reducing plastic pollution (Austin et al., 2018). Additionally, species of *Geobacter* have demonstrated the ability to detoxify pollutants ranging from heavy metals to radioactive compounds by using these substances as electron acceptors during metabolism, thereby converting them into less harmful forms (Lovley and Coates, 1997). Notably, *Geobacter* species have been extensively studied for their capacity to bioremediate uranium-contaminated groundwater by reducing uranium to a less soluble state (Korsman et al., 1992).

In the context of water purification, algal species such as *Chlorella* and *Spirulina* play a crucial role by absorbing nutrients, heavy metals, and other pollutants. These microalgae can effectively remove excess nitrogen and phosphorus, preventing harmful algal blooms and improving water quality (Olguín, 2012). Furthermore, extremophilic archaea belonging to the *Halobacteriaceae* family thrive in highly saline environments, such as industrial waste streams. These archaea have been successfully employed in bioreactors for the treatment of high-salinity wastewater, where they degrade organic contaminants and reduce overall pollutant load—an application particularly valuable in industries like petrochemical processing, where conventional treatment methods are ineffective (Oren, 2010).

## Challenges and future direction

Extremophiles offer great promise in many applications including agriculture, biofuels, pharmaceuticals and food processing. These properties make them invaluable candidates for designing industrial processes operating at high temperature, pH, or salinity and provide much more stable, cheaper and efficient alternatives to conventional enzymes (Mesbah, 2022). However, several key limitations currently restrict their wider industrial adoption. For instance, many extremophiles exhibit slower growth rates and lower biomass yields than conventional microbial chassis, and isolating and cultivating them in laboratory or industrial settings is often complicated by their highly specialized growth requirements (Babu et al., 2015) and These factors collectively contribute to a gap between laboratory-scale success and industrial scalability (Espina et al., 2021). To address these limitations, advances in metagenomics and bioinformatic analyses have provided researchers with the means to access extremophile genes without cultivation, partially overcoming the limited genetic tractability of many extremophiles. This has enabled the discovery of extremozymes with cross-sector industrial applications (Zhu et al., 2020). Genetic engineering and synthetic biology are also being used to transfer extremophilic traits to more easily cultivable microorganisms, improving scalability and cost-efficiency (Zhu et al., 2020). Furthermore, advances in bioreactor technology enable better control of the extreme conditions needed for cultivating extremophiles, potentially reducing production costs and increasing enzyme yields (Zhu et al., 2020).

In the future, extremophiles are expected to become much more useful in sustainable technologies, such as bioremediation, waste management, and renewable energy production (Swaminaathan et al., 2024). They also offer opportunities for developing new tools in drug discovery and food preservation, particularly through the production of stable bioactive compounds and enzymes (Jimenez et al., 2024). More extremophiles are now being used in the production of bioactive compounds and natural preservatives (Narayanan et al., 2024). In the future, extremophiles may play a key role in developing carbon-neutral or sustainable industrial processes due to their resilience and versatility in addressing global sustainability challenges, human well-being, as well as food security challenges (Chettri et al., 2021). In parallel, the integration of genome-scale metabolic modeling with synthetic circuit design can guide rational pathway optimization and regulatory control. Finally, process-level innovations in bioreactor design and operation will be critical to compensate for slower growth rates and enable efficient large-scale implementation.

## Conclusion

Extremophiles are diverse microorganisms capable of thriving in conditions lethal to most life forms, such as high or low temperatures, extreme salinity, acidity, alkalinity, radiation, or pressure. Found across all three domains of life, they possess unique metabolic, structural, and genetic adaptations—such as specialized enzymes, stress-response proteins, and efficient DNA repair systems—that enable survival under intense stress. Their evolutionary roots trace back to the Last Common Ancestor, which likely lived in hot, anaerobic conditions, suggesting that early life on Earth was thermophilic. Comparative genomics and metabolic modeling have revealed how extremophiles optimize energy use, genome structure, and metabolic networks to adapt to their habitats. In biotechnology, extremophiles are emerging as next-generation production platforms due to their natural tolerance to extreme conditions, reducing contamination and process costs. Metabolically engineered extremophilic species are used to produce valuable compounds like bioplastics, amino acids, and organic acids. Ecologically, extremophiles influence global cycles by fixing carbon, regulating methane emissions, degrading plastics, and detoxifying pollutants. Their resilience not only deepens understanding of life’s adaptability but also offers sustainable solutions for industrial production, bioremediation, and climate change mitigation. With continued study of their remarkable survival mechanisms, we may unlock solutions to global problems through these hardy lifeforms.
